# RankerGUI: A Computational Framework to Compare Differential Gene Expression Profiles Using Rank Based Statistics

**DOI:** 10.3390/ijms20236098

**Published:** 2019-12-03

**Authors:** Amarinder Singh Thind, Kumar Parijat Tripathi, Mario Rosario Guarracino

**Affiliations:** 1 High-Performance Computing and Networking Institute, National Research Council of Italy, Via P. Castellino, 111, 80131 Napoli, Italy; 2 Department of Medicine, Immunology and Allergy Unit, Karolinska Institutet, 171 76 Stockholm, Sweden; parijat24@gmail.com

**Keywords:** rank based statistics, gene expression comparison, transcriptomics data integration, next-generation sequencing, RNA-seq, microarray, web applications

## Abstract

The comparison of high throughput gene expression datasets obtained from different experimental conditions is a challenging task. It provides an opportunity to explore the cellular response to various biological events such as disease, environmental conditions, and drugs. There is a need for tools that allow the integration and analysis of such data. We developed the “RankerGUI pipeline”, a user-friendly web application for the biological community. It allows users to use various rank based statistical approaches for the comparison of full differential gene expression profiles between the same or different biological states obtained from different sources. The pipeline modules are an integration of various open-source packages, a few of which are modified for extended functionality. The main modules include rank rank hypergeometric overlap, enriched rank rank hypergeometric overlap and distance calculations. Additionally, preprocessing steps such as merging differential expression profiles of multiple independent studies can be added before running the main modules. Output plots show the strength, pattern, and trends among complete differential expression profiles. In this paper, we describe the various modules and functionalities of the developed pipeline. We also present a case study that demonstrates how the pipeline can be used for the comparison of differential expression profiles obtained from multiple platforms’ data of the Gene Expression Omnibus. Using these comparisons, we investigate gene expression patterns in kidney and lung cancers.

## 1. Introduction

Gene expression profiling provides an opportunity to explore the unique characteristics of biological states or phenotypes. With the availability of huge amounts of gene expression data in public repositories (The Library of Integrated Network-Based Cellular Signatures, ArrayExpress, Gene Expression Omnibus), it is possible to obtain the expression data for the comparison of studies across different experimental conditions. Comparative studies help in characterizing experimental conditions by their expression patterns, which in turn leads to the understanding of underlying transcriptional responses in diseases, drugs, gene perturbations’ effects, and complex interaction networks within genes and associated pathways. However, the small number of biological samples used in the experiment is a hindrance to expression analysis. Combining data from various existing research can make conclusions more reliable and generalizable. Therefore, data integration is a crucial step towards gaining new perspectives from big data produced by various independent studies intended to address similar biological problems.

Expression microarrays and next-generation sequencing (NGS) technologies are frequently used to study gene expression on a genome-wide scale. Nevertheless, complications appear when the different experimental setup adopts distinct protocols and technological platforms to compare cross-platform gene expression data. In such scenarios, to make data comparable to each other, researchers have focused on the development of rank based methods. In the last decade, researchers have successfully carried out drug–drug, drug–disease, and drug–target association studies through gene signature comparison to indicate potential drug repositioning [1,2]. Researchers have utilized a rank based scoring metric of gene expression signatures to calculate the similarity between the whole transcriptomes [2,3,4,5]. Rank based techniques were intended to avoid the use of cut-offs (such as a significant *p*-value and fold change), which select certain genes while excluding others arbitrarily. The OrderedList algorithm [6], for instance, focuses on using a similarity score to assess whether two lists have a significant similarity. The ArraySolver algorithm [7] compares microarray expression data using the Wilcoxon signed-rank test. The gene set enrichment analysis (GSEA) [3] and GOrillaalgorithms [8] rank genes by fold change to estimate gene sets’ enrichment. The algorithm of rank–rank hypergeometric overlap (RRHO) [9] compares lists of genes ranked by fold changes and focuses on visual representations of the similarities and dissimilarity between them.

**Figure 1:**
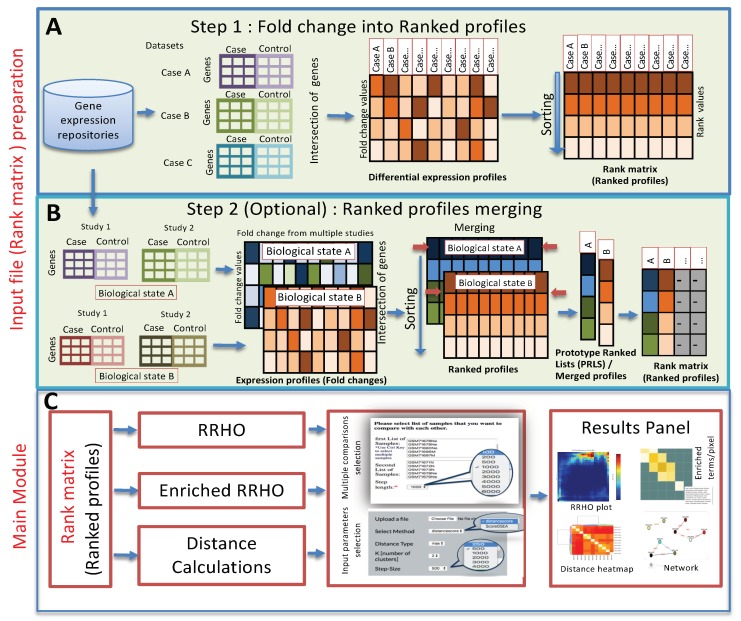
Outline of the pipeline. (**A**) The initial step is a preparation of the input file, which involves the collection of differential expression profiles for various comparisons among each other. Differential expression profiles are the first input to RankerGUI, which converts the fold change values into ranks called ranked profiles (ranked matrix) (note: the RankerGUI pipeline does not support differential expression analysis). (**B**) Step 2 merges the differential expression data from multiple individual studies, intended for the same biological state. (**C**) The main module has three different options, i.e., rank–rank hypergeometric overlap (RRHO), enrichment RRHO, and distance calculations.

Recently, many ranked based new tools have been developed for the comparison of differential expression profiles; some of these are Mode of Action by NeTwoRkAnalysis (MANTRA) (http://mantra.tigem.it) [10], parametric gene set enrichment analysis (PGSEA) [11], Gene Expression Signature [12], and RRHO [9]. RRHO and MANTRA are web applications without the support for the integration of gene expression profiles from multiple studies. The PGSEA and GeneExpressionSignature R packages integrate and analyze the expression data from multiple studies, but do not support the graphical user interface. Since, existing tools either lack the opportunity to support the integration of differential expression profiles or require programming skills, both of which put limits on the biologist in analyzing expression data obtained from multiple studies. To overcome these limitations, we developed a web based computational pipeline with a graphical user interface named “RankerGUI”. The rank based statistical module of the pipeline performs the analyses, to investigate the strength, patterns, and bound of correlation among differential expression profiles obtained from multiple experiments. These analyses can be done using the RRHO and distance calculation module (Figure 1). The pipeline provides results from multiple comparisons in a very organized way, with interactive visualizations.

Further, this pipeline incorporates various steps for the integration (preprocessing) along with main analysis modules (Figure 1). Preprocessing includes the conversion of expression ratio/fold changes into ranked profiles and an optional step for merging the ranked profiles. However, the pipeline does not support differentials expression analysis (DEA) and raw quality control steps, but we provided the instruction links for DEA [13,14,15] in the pipeline documentation. The merging step is important in cases where the user wants to group the differential expression profiles obtained from different studies for the same biological state. For example, in the preparation of input for the Biological State A ranked matrix, one may be interested in weighting the contribution of each of the cell lines equally. In that case, the user can opt for the merging option. Merging is an independent step and can be added before any main module.

## 2. Results and Discussion

RankerGUI portrays a global pairwise picture of differences among multiple differential gene expression profiles. It shows the intensity level of the overlap and its position between two ranked lists. The pipeline is straightforward to use as a modular computational application. In the back-end, it integrates different Bioconductor packages for rank based expression data comparisons. RankerGUI compares multiple biological states and multiple samples using different ranked based statistical approaches with low computational complexity. Each functional module of RankerGUI is independent and can be connected to others in the analysis. It provides visualization for fast interpretation of the results. Different modules of RankerGUI can explore the strength, pattern, and bounds of correlations among the expression profiles. There are options to re-run the job (without submitting the input file again) by selecting and fine tuning several features: from a distance method to a distance type, from signature length to window size etc, many different analyses can be quickly and conveniently performed. The interactive RRHO plots visualize the upregulated or downregulated pathways between two or more differential gene expression profiles based on overlapping genes belonging to bins in two expression profiles. Enrichment can be done by using the “KEGG Pathways” tool [16].

Furthermore, in the network visualization module, a network can be created and interactively visualized starting from the distance matrix. The complexity of the network can be changed for the interpretation of results by selecting a different threshold for edge weights. The higher minimum threshold for the network from the RRHO module shows strongly correlated gene profiles and hides profile interactions below the threshold. Therefore, this module is useful when comparing gene expression profiles at a global level.

### Case Study

We employed RankerGUI to detect global expression patterns/behavior among differential expression profiles of different cancer types obtained from a cross-platform comparative analysis. The aim was to identify a set of genes that behaves differently in lung cancer with respect to kidney cancer. Further, we wanted to check the reliability of the results by comparing the expression behavior with the extensive gene expression data available from TCGA database. To begin, we downloaded the cancer expression datasets for lung neuroendocrine tumor (GSE1037, platform GPL962) [17,18], clear cell carcinoma of the human kidney (GSE781, platform GPL96) [19], and human squamous cell carcinoma of the lung (GSE3268, platform GPL96) [20] using GEO2Rand the limma package [21,22] from Gene Expression Omnibus (GEO). These datasets are extremely heterogeneous not only with respect to the cancer type and the number of samples (both control and disease samples), but also the platforms used to obtain the gene expression profiles. The dataset of the lung neuroendocrine tumor is comprised of 19 normal lung control and 59 lung cancer samples. The clear cell carcinoma dataset of kidney contains eight normal and nine cancer samples. However, the dataset of human squamous cell carcinoma includes five normal and five cancer samples. Further, we inspected the distribution of expression values in each dataset and carefully selected only those datasets that were appropriately normalized.

**Figure 2:**
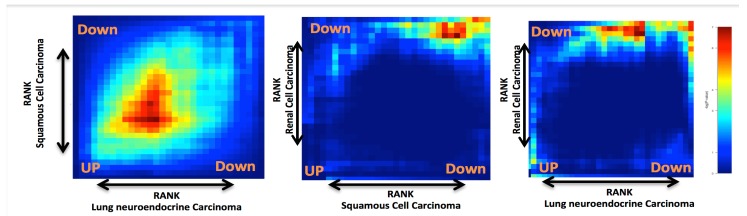
Heat maps show the rank rank hypergeometric overlap (RRHO): the red color along the diagonal axis shows a higher similarity between two gene expression profiles. The bottom left represents the similarity between the upregulated genes, while right upside represents the similarity between downregulated genes between two gene expression profiles. In (i), heat maps between squamous cell carcinoma and neuroendocrine lung carcinoma show more similarity as compared to the other two comparisons, i.e., (ii) between squamous cell carcinoma and renal cell carcinoma and (iii) between renal cell carcinoma and neuroendocrine lung carcinoma.

To carry out RankerGUI analysis, we selected only the commonly differentially expressed genes (intersection based on gene id) among different cancer types because the number of differentially expressed genes in each cancer type was different. From RRHO plots (Figure 2), we inferred that the behavior of genes in clear cell carcinoma of the kidney was different from the rest of the cancer types. This behavior was an interesting observation, as both neuroendocrine lung tumor and squamous cell carcinoma belong to a category of lung cancer, while both of these cancer are critically different from kidney cancer. We further narrowed down our search by taking a *p*-value cut-off 0.05 on commonly differentially expressed genes, which resulted in 874 genes. Gene ontology analysis for the Go-slim term associated with these 874 genes suggested the role of these genes in metabolic processes, cell death, communication, the cell differentiation process, protein binding, and catalytic activity with a significant *p*-value ≤ 0.05.

**Figure 3:**
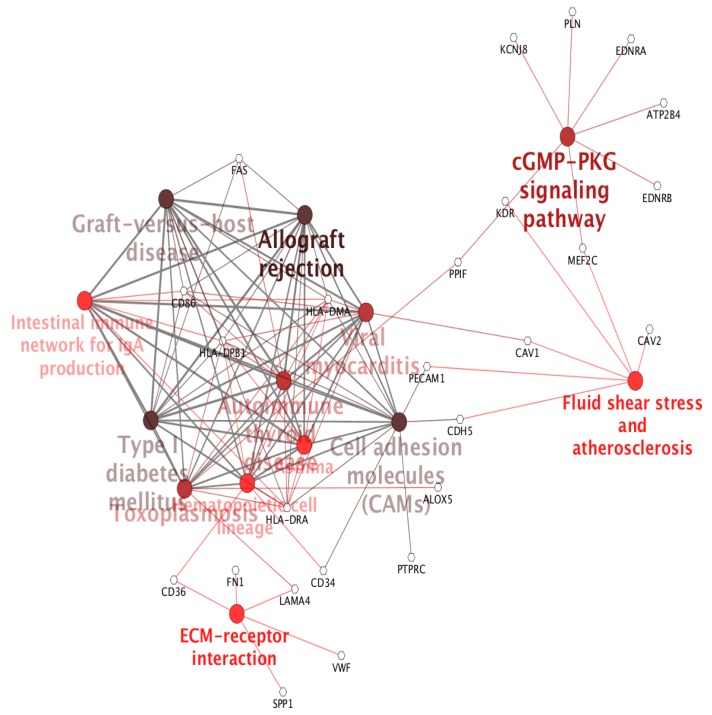
KEGG based pathway analysis of 86 genes reveals their significant role in different pathways, as shown in the figure, such as immune response pathways. A darker color represents more enrichment of the pathways, and genes are in the form of nodes.

**Figure 4:**
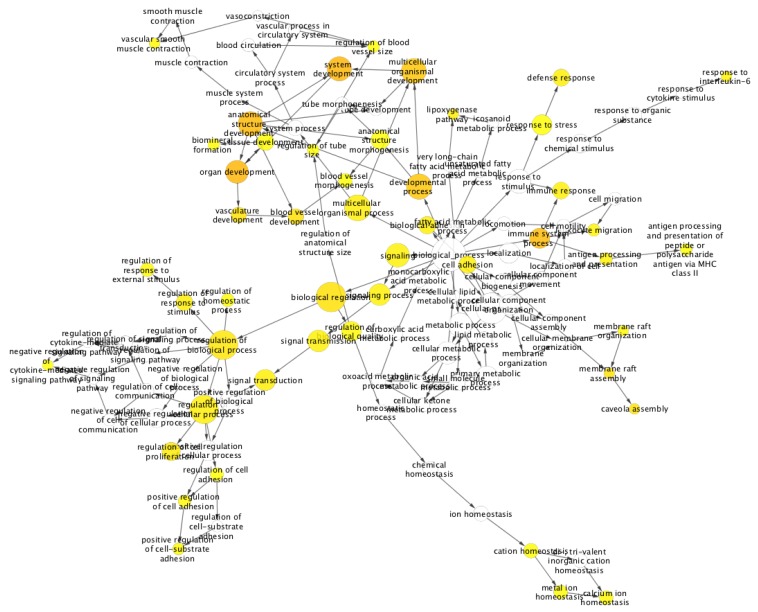
Gene ontology enrichment analysis of 86 genes, having distinct expression behavior in kidney and lung cancer. Here, orange and yellow nodes show the significantly strong association with the GO term (i.e., *p*-value ≤0.05) to those 86 genes, while white nodes represent a weak association with the GO term with slightly less significance than orange nodes and yellow nodes.

**Figure 5:**
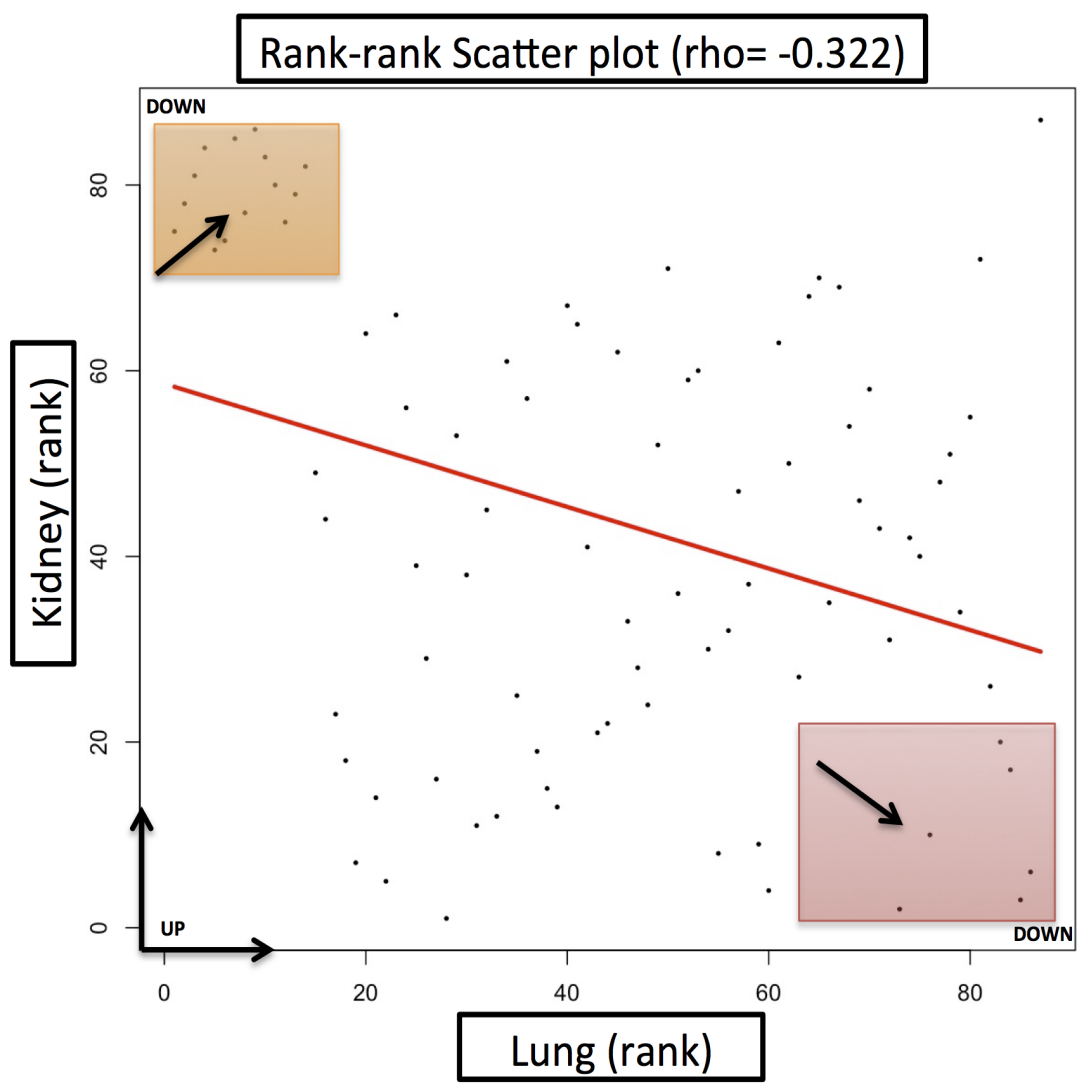
Scatter plot between lung and kidney cancer having a negative correlation of −0.332. We selected the top 20 distinctively behaving genes from both the up- and down-side shown in the figure in the form of small squares.

Additionally, to highlight the specific gene expression signature of lung versus kidney cancer, we selected 86 genes out of these common 874 differentially expressed genes based on the log 2-fold change cut-off ≥+1 and ≤−1. The logical basis for selecting this cut-off for differentially expressed genes was to find a subset of genes that demonstrated distinct expression behavior with respect to lung versus kidney cancer. We carried out the gene ontology and pathway enrichment network analysis on the common differentially expressed gene set using ClueGO [23]. It is a Cytoscape plugin and carries out a visualization of the non-redundant biological terms for big clusters of genes in a functionally grouped network. Taking a *p*-value cut-off ≤0.05, we obtained the gene ontology and KEGG pathway ( Figure 3) network for the differentially expressed gene sets. The gene ontology enrichment analysis for the biological processes of these 86 genes, as shown in Figure 4, suggested over-enrichment of biological regulation, signal transduction, anatomical structure development, the immune system, cell adhesion, regulation of cell adhesion, and regulation of cell proliferation with a significant *p*-value ≤ 0.05. Furthermore, some specific biological processes such as cytokine mediated signaling, response to interleukin-6, vascular smooth muscle contraction, response to stress, membrane raft and organization and assembly, caveola assembly, and antigen processing were enriched. Finally, these 86 genes showed a negative correlation of −0.332 between lung and kidney cancer. The scatter plot is provided in Figure 5.

**Figure 6:**
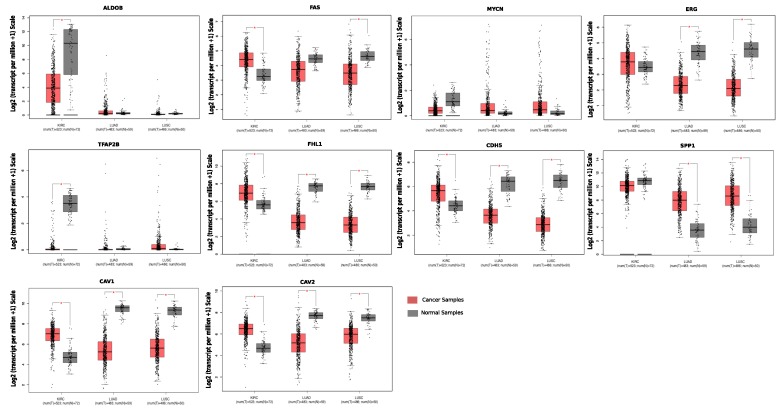
The figure shows the results of differential expression analysis from TCGA data in the form of a box plot for ALDOB, MYCN, TFAP2B, FHL1, CDH5, SPP1, CAV1, CAV2, FAS, and ERG genes. In each figure, the comparison of normal (grey) versus cancer (red) tissue is shown among three different cancer types, i.e., lung adenocarcinoma (LUAD), lung squamous cell carcinoma (LUSC), and kidney renal clear cell carcinoma (KIRC).

We also studied the differential expression behavior of our candidate genes with respect to the cancer data available in The Cancer Genome Atlas (TCGA). For this purpose, we used a web application platform, GEPIA [24]. It is an interactive web server for analyzing the RNA sequencing expression data of tumors and normal samples from the TCGA and the GTEx projects, using a standard processing pipeline. Using this web application, we carried out differential expression profiling of our target genes across lung adenocarcinoma samples (LUAD), lung squamous cell carcinoma (LUSC), and kidney renal clear cell carcinoma (KIRC) tumor samples and paired normal tissues. We also carried out the overall survival (OS) analysis based on the differential gene expression of our genes in lung and kidney cancer and plotted the Kaplan–Meier estimator using a log-rank test cut-off *p*-value ≤0.05, for the hypothesis test. For this purpose, out of these 86, we specifically selected the topmost genes (top left and bottom right corners in Figure 5), which portrayed distinctive behavior with respect to lung and kidney cancer. These genes were most probably a driving force behind the observed negative correlation between lung and kidney cancer. Table 1 provides the list of these genes with their differential expression value. The differential expression analysis results from TCGA data, as shown in Figure 6, coherently agreed with the results from our datasets. We studied the differential regulation patterns of ALDOB, MYCN, TFAP2B, FHL1, CDH5, SPP1, CAV1, and CAV2 in TCGA datasets of LUAD, LUSC, and KIRC cancer samples with respect to their controls.

Additionally, we included two more genes FAS and ERG (a proto-oncogene) from our list of 86 genes and carried out differential expression pattern analysis in the LUAD, LUSC, and KIRC datasets. From the TCGA analysis of these genes, we inferred that ALDOB and TFAP2B (the top two downregulated genes in the ranked list of kidney cancer) and MYCN were significantly downregulated in kidney renal cell carcinoma with respect to normal kidney tissue, while ALDOB, TFAP2B, and MYCN were upregulated in the case of LUAD and LUSC cancer samples with respect to the normal controls. Similarly, by analyzing the expression behavior of FHL1, CDH5, CAV1, and CAV2 genes from TCGA datasets of LUAD, LUSC, and KIRC, we observed that these genes were significantly downregulated in lung cancer, whereas upregulated in kidney cancer samples.

Our case studies showed the capability of the RankerGUI platform to explore and compare the differential gene expression data obtained from various platforms. RankerGUI quickly detected the different behaviors of the set of genes between lung and kidney cancer by utilizing differential gene expression profiles. Further, a similar behavior for the set of genes was observed from TCGA expression data comparison.

## 3. Method and Implementation

### 3.1. Method

RankerGUI is a graphical user interface to an integrated computational pipeline (Figure 1). It compares multiple differential expression profiles and characterizes interesting gene signatures. It also provides an option to convert differential expression values into ranked profiles, a pre-requirement step for the distance calculations and RRHO based analysis.

Details of the RankerGUI modules are defined below:

The differential expression into ranked profiles module sorts the differential expression profiles in descending order, providing a rank for each gene. These profiles are known as ranked profiles. This module is implemented in Python.

The merging of ranked profiles can be used to merge two or more differential expression profiles. This module uses an approach developed by Iorio et al. [2], which uses the Kruskal algorithm [25] strategy. The function executes multiple iterative steps, in which distances between all ranked profiles are calculated, and two closely related profiles are merged each time until only one merged ranked profile remains. This merged ranked profile is known as the prototype ranked list (PRL).

Spearman’s foot rule calculates the distance between all possible paired combinations of ranked lists. The description of Spearman’s foot rule [26] is as follows:

(1) $$D\left(x;y\right)=\sum_{i=1}^{N}\left|R_{\left(i,x\right)}-R_{\left(i,y\right)}\right|$$

In the above equation, D(x;y) is the distance between *x* and *y* ranked expression profiles with *N* genes, where $R_{\left(i,x\right)}$ is the rank of the $i^{\mathrm{th}}$ gene in ranked expression profile xand $R_{\left(i,y\right)}$ is the rank of the same gene ($i^{\mathrm{th}}$) in ranked expression profile y.

Then, using the Borda mean rule [27], it is possible to merge two closely matched rank profiles (ranked lists). For each iteration, these pairs of ranked profiles (ranked lists) are deleted from the existing data and substituted with a single merged profile.

Rank–rank hyper-geometric overlap analysis module: The RRHO plot highlights the correlational strength in the form of the $log_{10}$ transformed hypergeometric *p*-value between two differently expressed ranked profile. This module uses the “RRHO package” [9] from Bioconductor in R. Following are the three main steps of RRHO analysis:

(1) The RRHO algorithm uses the hypergeometric probability (defined in the following), which determines the enrichment between the overlapping set of genes. Two lists of bins (pixels) are defined based on step size and ranked list length following hypergeometric overlap. The iteration on each ranked list (for both lists of defined bins) measures the significance of overlapping genes using hypergeometric distribution functions. Bins are significant if the number of overlapping genes is more (or less) than expected by random chance. Finally, a hypergeometric *p*-value matrix is obtained for each list of bins.

Hypergeometric distribution function: Suppose *N* is the length of the ranked list (population size), *k* of which are successes, and a random sample drawn from *N* consists of *n* items (sample size), *u* of which are successes (overlapping genes). *k* and *n* are the rank in two rank lists at the particular step of iteration. Then, the hypergeometric probability is:

(2) $$h\left(u;N,n,k\right)=\frac{{(}_{u}^{k}){(}_{n-u}^{N-k})}{{(}_{n}^{N})}$$

(2) Further, *p*-values are transformed using log${}_{10}$. The positive sign shows that overlap is more than expected, and the negative sign shows that it is less than expected by chance. This representation shows the hypergeometric *p*-value matrix in the form of a heat map because significant bins are visible on the RRHO plot.

Pathway enriched rank–rank hypergeometric overlap analysis module: This module introduces interactive enrichment for each pixel (bin) of the RRHO plot, which is based on the cluster profile package of Bioconductor and d3.js library. This step extracts the list of overlapping genes in each pixel along with the hypergeometric *p*-value matrix. In the background, R scripts automatically fetch enrichment for overlapping genes for each pixel from different databases using the package clusterProfiler [28] and embed the enriched output into the interactive RRHO plot using d3.js. This module supports “KEGG”, “NCBI (Entrez)”, and “UniProt” gene id for enrichment analysis. Currently, RankerGUI supports enrichment for “Human”, “Rat”, “*Drosophila*”, “Zebra Fish”, and “Mouse”. This support will be further extended in the future.

Distances among ranked profiles: The distance between two ranked profiles is calculated using the most useful user defined gene signatures. For instance, the s-length signature contains lists of *s* genes from the ranked profile top ranked (*p*) and bottom ranked (*q*) genes.

(3) $$p=p_{1},p_{2},....p_{s}\;\;and\;\;q=q_{1},q_{2},....q_{s}$$

where *p* and *q* are the top and bottom ranked genes for ranked profile “*x*”.

The signature length is generally heuristically characterized. The pipeline offers two choices for distance calculations: average distance and maximum distance for enrichment score [2,12]. These distances are defined as the inverse total enrichment score (TES) [2], which is measured using the enrichment score (ES) of GSEA [3], which is based on Kolmogorov–Smirnov statistics. The ES represents the degree of over-representation of the genes in a gene set at the top or bottom of the ranked list of genes. The calculation of the ES is performed by walking down the ranked gene list, increasing a running-sum statistics if a gene belongs to the set, and decreasing it if the gene does not. The ES is the highest possible deviation detected during a walk from zero [3].

TES of the ranked profile “*x*” with respect to the ranked profile “*y*” is as follows:

(4) $$TES_{x,y}=1-\frac{ES_{y}^{p}-ES_{y}^{q}}{2}$$

where $ES_{y}^{p}$ is the enrichment score of “s” top-ranked genes of *x* with respect to the ranked profile of *y*, and similarly, $ES_{y}^{q}$ is the enrichment score of “s” bottom ranked genes of *x* with respect to the ranked profile of *y*. ES ranges between [−1, 1], while TES ranges in [0, 2]. An enrichment score closer to either −1 or one means more genes are at the bottom or top, respectively. TES measures the numbers of genes in a set p present at the top of the ranked profile *x*. The same definition is applicable to the set q for the bottom of the ranked profile. If both statements are true (e.g., same ranked profiles), the TES value will be zero. Two types of distances, as mentioned earlier, can be calculated in the following way:

Average enrichment-score distance:

(5) $$D_{avg}=\frac{TES_{x,y}+TES_{x,y}}{2}$$

Maximum enrichment-score distance:

(6) $$D_{max}=\frac{min(TES_{x,y}+TES_{x,y})}{2}$$

To obtain these distance score, RankerGUI employs the scoreGSEA and distance function [2,12] from the “GeneExpressionSignature” package [12].

Affinity propagation clustering module: In this module, we employ the apcluster package [29,30] from Bioconductor to run affinity propagation clustering to group biological and technical samples based on the similarity of their gene signature. This algorithm iteratively searches for optimal clustering by maximizing an objective function called net similarity.

Network visualization module: This module uses Cytoscape JavaScript [31], which provides an application user interface (API) for interactive graphs embedded in the web user interface. The user input file is required in the form of the distance matrix (details of the input file format are available on the documentation page of the pipeline and from the example file). The network module also links to the distance and RRHO module, and distances of the input are calculated as the average score from the distance module and log10-transformed hypergeometric P-values from the RRHO module.

### 3.2. Web Server

The RankerGUI web server uses the Mac platform with the Apache web server. The front-end interface is built using the HyperText Mark-up Language (HTML5), Hypertext Processor (PHP Version 5.3.28), JavaScript, and Cascading Style Sheets (CSS3). The front-end provides options for easy job submission and multiple comparison choices. The Cytoscape JavaScript (Version-3) [31] library was used for interactive network visualization. After job completion, the web server sends a link to an HTML page to the user’s email address. RankerGUI saves the computational results of all users on the server for at least one month. At the back-end, the web server is also connected to a MYSQL (5.5) database to maintain the incoming jobs and to execute in a queue based manner. In the case of multiple tasks for the particular module, it saves jobs in the database and runs them one by one. The “Documentation” section on the web server provides more information about features and uses. The web server application is freely available along with example files for each module at http://watson.na.icar.cnr.it/rankergui/index.php.

## 4. Conclusions

RankerGUI provided a rich set of features to explore differential gene expression data and capture the signature or patterns from different experimental conditions using heterogeneous data. It allowed the user to use rank based approaches for differential expression profiles’ comparison, i.e., RRHO and distance calculations, straightforwardly. A further output of these two modules was used for network construction and affinity propagation. Merging of data from multiple individual studies of the same biological state could be done. For a better interpretation of the results, network and RRHO modules were integrated with d3.js and cytoscape.js based interactive visualization. For example, there was the possibility to analyze the complex network quickly by changing the thresholds of the edge weights. RRHO plots also provided enrichment for each pixel and could be visualized interactively. Our case study captured the patterns from RRHO plots for the list of signature genes whose expression behavior was distinct in different cancer types. This multi-platform study helped to predict a set of genes, which can act as a driving force to distinguish clear cell carcinoma of the kidney from primary lung cancers.

## Figures and Tables

**Table 1:** List of selected top 20 genes, which show distinctive differential expression behavior in lung and kidney cancer.

Gene Name	Fold Change (FC) (Lung Cancer)	Fold Change (FC) (Kidney Cancer)	Description
ALDOB	1.13	−7.01	aldolase, fructose-bisphosphate B
TFAP2B	1.17	−4.16	transcription factor AP-2 beta
AZGP1	1.23	−3.95	alpha-2-glycoprotein 1, zinc-binding
PC	1.05	−2.39	pyruvate carboxylase
PPM1H	1.01	−2.37	protein phosphatase, Mg2+/Mn2+ dependent 1H
GGH	1.24	−2.35	gamma-glutamyl hydrolase
FOXI1	1.05	−2.26	forkhead box I1
MYCN	1.02	−2.10	v-myc avian myelocytomatosis viral oncogene neuroblastoma derived homolog
UCHL1	1.47	−1.70	ubiquitin C-terminal hydrolase L1
TUBB2A	1.15	−1.52	tubulin beta 2A class IIa
PPIF	1.03	−1.32	peptidylprolyl isomerase F
SPP1	2.29	−1.15	secreted phosphoprotein 1
PFN2	1.2	−1.06	profilin 2
PDHA1	1.21	−1.02	pyruvate dehydrogenase (lipoamide) alpha 1
CALCRL	−2.04	1.00	calcitonin receptor like receptor
CDH5	−2.08	1.71	cadherin 5
CAV2	−2.1	1.86	caveolin 2
PMP22	−2.11	1.97	peripheral myelin protein 22
FHL1	−2.52	3.09	four and a half LIM domains 1
CAV1	−3.39	2.92	caveolin 1

## Abbreviations

GSEA	Gene set enrichment analysis
KIRC	Kidney renal clear cell carcinoma
LUAD	Lung adenocarcinoma
LUSC	Lung squamous cell carcinoma
PRL	Prototype ranked list
RRHO	Rank–rank hyper-geometric overlaps
